# Total and Free Sugars Consumption in a Slovenian Population Representative Sample

**DOI:** 10.3390/nu12061729

**Published:** 2020-06-09

**Authors:** Nina Zupanič, Hristo Hristov, Matej Gregorič, Urška Blaznik, Nataša Delfar, Barbara Koroušić Seljak, Eric L. Ding, Nataša Fidler Mis, Igor Pravst

**Affiliations:** 1 Nutrition Institute, Tržaška cesta 40, SI-1000 Ljubljana, Slovenia; nina.zupanic@nutris.org (N.Z.); hristo.hristov@nutris.org (H.H.); 2 Department of Food Science and Technology, Biotechnical Faculty, University of Ljubljana, Jamnikarjeva 101, 1000 Ljubljana, Slovenia; 3 National Institute of Public Health, Trubarjeva 2, SI-1000 Ljubljana, Slovenia; matej.gregoric@nijz.si (M.G.); urska.blaznik@nijz.si (U.B.); natasa.delfar@nijz.si (N.D.); 4 Computer Systems Department, Jožef Stefan Institute, 1000 Ljubljana, Slovenia; barbara.korousic@ijs.si; 5 Department of Nutrition, Harvard Chan School of Public Health, Boston, MA 02115, USA; eding@post.harvard.edu; 6 Department of Gastroenterology, Hepatology and Nutrition, University Children’s Hospital, University Medical Centre Ljubljana, Bohoričeva ulica 22a, SI-1000 Ljubljana, Slovenia; natasa.fidler@kclj.si; 7 VIST—Higher School of Applied Sciences, Gerbičeva cesta 51A, SI-1000 Ljubljana, Slovenia

**Keywords:** free sugars, sugars intake, dietary intake, 24-h recall, nutrition survey, public health, EU Menu, Slovenia

## Abstract

Excessive free sugars consumption is associated with poor health outcomes. Thus, the World Health Organization (WHO) recommends limiting free sugars intake to no more than 10% of total energy intake. To evaluate current intakes of dietary sugars and monitor the adherence to the guidelines, the objective of this study was to comprehensively assess total and free sugars consumption of different age groups within the Slovenian population. The Slovenian national food consumption survey SI.Menu 2017/18 was conducted on representative samples of adolescents (10–17 years), adults (18–64 years), and the elderly (65–74 years) using two non-consecutive 24-h dietary recalls. The analyses were carried out on a sample of 1248 study participants. Free sugars content in food was estimated based on previously established databases. The population weighted median free sugars intake accounted for 10.1% of total energy intake (TEI) among adolescents, 6.4% among adults, and 6.5% in the elderly population. Both total and free sugars consumption in the percentage of TEI were higher among women than men, in participants with lower education, and those with higher family net income. The main sources of free sugars in adolescents were beverages, cakes, muffins, pastry, and dairy products; for adults and the elderly, the key sources of free sugars were beverages, cakes, muffins, pastry, and sugars, honey, and related products. A total of 56% of adolescents, 84% of adults, and 81% of the elderly population adhered to the WHO free sugars guidelines. Additional measures will be required to further decrease free sugars consumption among the teenage population, in which dietary patterns are still of greatest concern.

## 1. Introduction

Sugar is a highly palatable and a widely accessible commodity. Due to its rewarding effect on the brain, eating behavior is frequently decoupled from the feeling of hunger, leading to frequent overconsumption of sugary foods and drinks [1,2]. Higher intake of dietary free sugars, especially in the form of soft drinks, is a well-documented risk factor for obesity and oral disease [3,4], while several studies suggested a link with other metabolic diseases [5,6] and even some forms of cancer [7]. Although the associations of free sugars consumption with obesity are often claimed to be merely a result of increased energy intake, studies have emphasized the incomplete energy compensation for energy delivered from sugars, particularly when ingested in liquid form [8,9,10]. Adverse changes in serum lipids and blood pressure were observed in relation to frequent sugar consumption without reported differences in weight change [11].

Mounting evidence of the detrimental effects of free sugars overconsumption has led the World Health Organization (WHO), the British Scientific Advisory Committee on Nutrition (SACN), the European Society for Pediatric Gastroenterology Hepatology and Nutrition (ESPGHAN), and several other public health organizations to recommend limiting free sugars intake to less than 10% of the total energy intake (TEI), and preferably below 5% for additional health benefits [12,13,14]. 

According to the WHO, free sugars include all sugars added to food either by the manufacturer, the cook, or the consumer, as well as sugars naturally present in honey, syrups, fruit juices, and fruit juice concentrates [12]. Our previous research showed that more than half of all pre-packaged products on the Slovenian market contain free sugar ingredients, although the sales data revealed that within pre-packed foods, the majority of free sugars is sold as chocolate and sweets, soft drinks, and biscuits [15,16]. Focused solely on pre-packaged products, previous studies potentially missed some important categories of sugar containing foods: home-baked sweets, restaurant meals, and other non-branded sweet products. 

The most recent available data on dietary habits among different age groups in Slovenia are scarce and outdated [17,18], complicating the evaluation of the adherence to dietary guidelines on free sugars intake. Thus, the aim of the present study was to investigate dietary intake of total and free sugars among different age groups of Slovenian population, identify main sources of free sugars, and to evaluate demographic and lifestyle parameters that correlate with higher consumption of free sugars. 

## 2. Materials and Methods

### 2.1. Study Design and Population

The cross-sectional Slovenian national food consumption survey, SI.Menu 2017/2018, was administered between March 2017 and April 2018 and followed the European Food Safety Agency (EFSA) Guidance on European Union (EU) Menu Methodology [19]. The complete study design is described in detail elsewhere [20]. In brief, the participants were Slovenian residents, stratified into three age groups: adolescents (10–17 years old), adults (18–64 years old), and the elderly (65–74 years old). A total of 2280 individuals were selected using the Central Register of Population of Slovenia according to age, size and type of household, and place of residency (to cover all 12 statistical regions of Slovenia). Individuals living abroad, and institutionalized, ill, or people with disabilities were excluded from the study. Selected individuals were invited to join the study by an invitation letter and, within two weeks, they were visited by one of the trained interviewers who further explained the survey procedure and determined the eligibility of the participant. The response rate was 62.2% (*n* = 1319).

The survey protocol was registered and accepted by the National Medical Ethics Committee (KME 0120-337/2016). All participants were informed about the details of the survey and thereafter signed a written informed consent form. For the participants younger than 18 years of age, written consent was also signed by the parent(s) or legal guardian(s). 

### 2.2. Dietary Assessment Methods

#### 2.2.1. General Questionnaire and Anthropometric Measurements

At the first face-to-face interview, participants completed a general questionnaire consisting of questions on socio-demographic and socio-economic determinants such as household size, marital status, education, and monthly income of the household. Participants were asked to provide self-reported physical activity levels, which were later converted to the International Physical Activity Questionnaire (IPAQ) score as described by Craig et al. [21]. Participant’s weight and height were measured at the end of the first interview, upon which body mass index (BMI; kg/m^2^) was calculated. The cut-off points for overweight and obesity were set at 25 kg/m^2^, except for adolescents, where sex/age adjusted cut-off points (>+1 SD) were used [22,23].

#### 2.2.2. 24-h Dietary Recall

The interviewers performed two 24-h dietary recalls on two non-consecutive days at the participant’s home. The second recall was repeated between 7 days and 3 weeks after the first, but had to be performed on the same day of the week as the first (71% of all recalls were carried out on working days and 29% on weekends). Approximately 87% of second 24-h recalls were repeated within 7 days after the first recall, while the rest were completed within 7–21 days. During the recall, participants were asked to reproduce the data on food and beverages consumed during the preceding day following a daily meal timeline. Portion sizes were estimated using a nationally adjusted picture book that contained 46 pictures of different food products or simple recipes, with each one photographed in 6 different portion sizes. Validation of the book was performed in May 2015 at Biotechnical Faculty, University of Ljubljana, Slovenia [20]. 

All answers were collected with the Open Platform for Clinical Nutrition (OPEN) app, designed by the Jozef Stefan Institute (Ljubljan, Slovenia) specifically for the purpose of SI.Menu data collection. To ensure smooth interview procedures, the app is fully functional with no Internet access, with the data stored to the server whenever Internet access is available. The OPEN app is an extension of the national nutrient database OPEN [24], which maintains food composition data of generic foods and provides a list of ingredients for traditional and other recipes frequently used in Slovenia. When needed, missing data was completed with information from other European and United States Department of Agriculture (USDA) food composition databases [25]. 

### 2.3. Assessment of Nutrient Intake

All foods and beverages reported during 24-h recalls were assigned the appropriate energy and nutrient contents based on data from the Open Platform for Clinical Nutrition (OPEN) [24] and previously established database with free sugars content in foods [16]. In the OPEN database the energy content of foods and beverages is calculated based on macronutrient, alcohol, and dietary fiber content. When information on dietary fiber content was available, total energy value was calculated as total available energy, i.e., excluding non-digestible fiber. In certain foods and beverages, in which very low content of dietary fiber was expected, total energy content was calculated as 17 kJ per gram of total carbohydrates. To enable accurate nutrient profile formation for more complex foods and dishes, a disaggregation method was applied based on the recipes provided by the subjects, when applicable, or traditional recipes collected in OPKP. To differentiate between pre-packaged and non-packaged foods, each food/beverage item was determined as branded or non-branded and allotted to one of the 17 predefined categories, which were further divided into 96 subcategories. The categorization system was adapted from Dunford et al. [26], with the additional subcategories added only for non-packaged and home-cooked foods. 

### 2.4. Exclusion Criteria and Assessment of Under- and Over-Reporting

The exclusion criteria were determined based on three different parameters: (1) incomplete anthropometric data (*n* = 10), (2) incomplete 24-h recall data (*n* = 38), and (3) under- and overreporting (*n* = 49). Altogether, data from 97 individuals were excluded from further analyses. Demographic characteristics of the final study sample are presented in Table 1.

Under- and over-reporting were assessed using the cut-off points method initially described by Goldberg et al. [27] and further adapted by Black et al. [28]. The method is based on the ratio of reported daily energy intake and basic metabolic rate (BMR). BMR was calculated based on sex, age, body height, and body weight using the method described by Harris et al. [29] and adapted by Roza and Shizgal [30]. The calculated cut-off points for 24-h recalls for under- and over-reporting were 0.41 and 2.46, respectively. Participants reporting energy intakes of less than 500 kcal were also excluded from the analyses.

### 2.5. Statistical Analyses

All statistical analyses were conducted using STATA version 15.1 (StataCorp LLC, Coledge Station, TX, USA). Total energy intake was adjusted according to the nutrient residual (energy-adjusted) method [31]. Energy and nutrient intake estimates were not normally distributed; thus, the normalization was performed using log transformation upon need. As the 24-h recalls measured only short-term consumption pattern, the two recalls were combined to estimate the habitual sugars consumption and reduce day-to-day variability using the multiple source method (MSM) [32]. The method addressed the partitioning of variance and adjusted for between- and within-person variations to calculate the usual intake. However, it was used under the assumption that all survey participants were potential consumers of total and free sugars. The final data set was weighted with iterative proportional fitting [33] for deviances in age, sex, and region, using census data from the 2017 reference population. 

Descriptive characteristics (mean, median, proportions) are presented for age cohorts and for different socio-demographic-, anthropometric-, and individual-based variables within each age group. Linear and logistic regression analyses were used to determine the significant differences between different sub-populations in terms of free sugars consumption based on TEI. The unadjusted means of free sugars in %TEI were determined by sex, place of living, BMI, and IPAQ levels for all age groups, education, income for adults and elderly, and employment status for adults only. The prevalence of consumption of less than 10% TEI in free sugars was calculated separately for all age groups and correlation determined with socio-demographic, anthropometric, and lifestyle parameters. Multivariable logistic regression analysis was used to determine independent predictors for consumption of free sugars in amounts larger than 10% TEI. Model parameters were estimated by the maximum likelihood method. Odds ratios (ORs) with 95% confidence intervals (CIs) were used as a measure of relative risk. Differences were considered significant at *p* < 0.05.

## 3. Results

In adolescents, the energy-adjusted median habitual total daily sugars intake (free plus intrinsic) was 74.2 g, accounting for 18.1% (95% CI: 17.3, 19.0) TEI, whereas median free sugars intake, as defined by the WHO, was 39.3 g or 10.1% (95% CI: 9.55, 10.66) TEI. The intake of total and free sugars in adults was lower, at 59.3 g or 14.6% (95% CI: 13.8, 15.3) TEI and 22.6 g or 6.4% (95% CI: 6.0, 6.9) TEI, respectively. In the elderly, the median total sugars was 65 g (15.9% (95% CI: 15.1, 16.6) TEI) and median free sugars 21.8 g or 6.5% (95% CI: 5.7, 7.4) TEI (Table 2). Women had a higher relative intake of total sugars in all age groups by approximately 5% of TEI. The in-between gender trend was the same, although the difference less pronounced, in habitual free sugars consumption. The percentage of carbohydrates consumed in the form of free sugars was the highest among adolescents (19.5%), whereas in adults and the elderly, free sugars accounted for 13.8% of all carbohydrates consumed.

Across all age groups, teenage girls had, on average, the highest percentage of calories consumed in the form of free sugars and had the lowest percentage of participants adhering to the general WHO recommendations for free sugars intake (46.9%). Conversely, the lowest consumption of free sugars was observed in adult men, whose average intake was as low as 5.6% of TEI. The adherence to the WHO guidelines among adult men was 88.8%.

In all age groups, women consumed a significantly higher proportion of their daily caloric intake in the form of free sugars compared to men (Table 2). In adults, the difference was significant between those with no university degree, with a higher consumption of free sugars compared to participants with a higher education (7% TEI compared to 5.6% TEI, respectively). Higher free sugars intake was also observed in adults living in families with higher household net income (Table 3; supplementary Table S1).

In Table 4, foods are organized into categories and subcategories according to the modified Dunford classification system [26] and presented in relation to their contribution to the total intake of sugars. Only relevant food groups are shown; data for all food categories can be found in Table S2. The main sources of total and free sugars differed among different age groups and, to a lesser degree, between the sexes. In adolescents, the three main sources of free sugars were beverages (32.7%), bread and bakery products (21.2%), and dairy (10.7%). The first two categories were also major sources of free sugars in adults at 34.1%, and 20.0%, respectively, whereas the third-most common source of free sugars were sugars, honey, and related products (14.0%). In the elderly, the main contributors to free sugars intake were also beverages (41.1%); sugars, honey, and related products (21.8%); and bread and bakery products (20.2%). In the elderly, around 10% of the total free sugars intake was from dairy products, whereas adolescents also consumed around 9.2% through confectionary. 

Among beverages, soft drinks and fruit and vegetable juices were the largest contributors to free sugars intake across all age groups (Table 4). In adolescents and the elderly, the contribution of fruit and vegetable juices was even greater than that of soft drinks, whereas adults reported consuming more free sugars in form of soft drinks than juices. Among the elderly, a larger proportion of free sugars came from honey (12.8%) compared to adolescents who consumed honey in small quantities (1.8%). In the category of bread and bakery products, cakes, muffins, and pastry accounted for the majority of free sugars consumed in the category (11–13.4%), followed by biscuits (6.3–8.5%). The category of fruit and vegetable products was also an important contributor to free sugars intake among all age groups, with a quite high intake of fruit jams and marmalades, particularly among the elderly (5.7–8.3%). 

We also examined the proportion of participants who met the WHO recommendations (advising that free sugars should represent no more than 10% of the TEI). Overall, 56.0% (95% CI: 51.4, 60.4) of adolescents, 82.1% (95% CI: 77.8, 85.8) of adults, and 85.6% (95% CI: 81.9, 88.6) of the elderly adhered to these guidelines (Table 5). When examining the effects of different socio-economic and lifestyle parameters in terms of free sugars intake (%TEI) prevalence, statistically significant differences were observed between men and women in the adolescent and adult age groups. As mentioned earlier, in both age groups, men consumed significantly less free sugars compared to women and a significantly higher percentage of men adhered to the upper limit of free sugars intake. The difference between adolescent boys and girls was especially pronounced, with 68.9% and 42.6%, respectively. Although statistically not significant, the same trend was observed among the elderly (Table 5). Besides sex differences, among adults, free sugars intake also differed with respect to family net income. Odds ratios were significantly lower for the population with lower household net incomes (OR: 0.45; 95% CI: 1.04, 4.72) compared to the other group. Conversely, place of living, level of education, BMI, IPAQ score, and employment status were found to be non-significant predictors in the prevalence of free sugars intake as specified in the guidelines. Adherence to the conditional recommendation (free sugars less than 5% of TEI) is presented in Table S3. The prevalence of the population adhering to the advanced recommendation was 13.4% (95% CI: 10.3, 16.4) among adolescents, 44.0% (95% CI: 38.9, 49.1) among adults, and 47.1% (95% CI: 42.3, 51.9) among the elderly. Notably higher adherence to the 5% TEI recommendation was observed in male participants, regardless of age.

## 4. Discussion

The results presented here provide the most recent overview of the estimated intakes and sources of total and free sugars in the Slovenian population. The median intake of total sugars was 18.1% TEI in adolescents, 14.6% TEI in adults, and 15.9% TEI among the elderly, whereas median free sugars intakes ranged from 10.1% TEI in adolescents, to 6.4% TEI and 6.5% TEI in adults and the elderly. The results indicated that habitual consumption of total and free sugars is the highest among 10–17-year-olds, which is in line with other studies, although the reported intakes are somewhat lower compared to the other European countries. In the U.K. National Nutrition Survey (2014–2016), teenagers were reported to consume on average 13% TEI from free sugars [34]. The results from the Dutch National Food Consumption Survey occurring 2007–2010 were even more alarming, with the reported median daily intake of free sugars in adolescents being as high as 17.6–19.8% TEI [35]. Conversely, according to the Spanish Anthropometry, Intake and Energy Balance (ANIBES) study carried out in 2013, Spanish children (9–12 years) and adolescents (13–17 years) tend to consume similar proportion of energy in free sugars (9.8% and 10% TEI, respectively), as was observed in the present study [36]. 

In 2016, Newens and Walton [37] published a review of total and added sugars consumption surveys across the world, reporting that total sugars consumption for adolescents ranged from 15.4% TEI in Italy to 29.6% TEI in Germany. With an 18.1% TEI, the average intake of total sugars for Slovenian girls and boys aged 10–17 years is in line with the reported global estimates, albeit on the lower end. Free sugars consumption estimates for Slovenian adolescents are lower than the reported range of 12.4–16% TEI for added sugars across six different countries. Although “added” and “free sugars” are not completely interchangeable terms, definition of free sugars is broader and thus a hypernym of added sugars [38]. Hence, the value for free sugars intake should be equal to or higher than added sugars value. 

A study on nutritional habits among Slovenian adolescents (14–17 years) published in 2012 was the first cross-sectional nutritional study in Slovenia to include data on free sugars intake. The results can thus serve as a reference point to observe the trends in free sugars consumption, although the comparisons should be conducted with caution as the methodology used in the previous study (food frequency questionnaire) is not directly comparable to the present method (24-h recall). In 2012, Slovenian teenage girls and boys consumed 16–17% TEI in the form of free sugars [17], whereas the intake observed in our study was 10.1% TEI. This difference could be an encouraging result of active campaigns and education programs informing people about the negative effects of excessive free sugars consumption, especially in the form of soft drinks, but could also arise (at least in part) from methodological differences and more frequent selective under-reporting. Studies showed that foods perceived as generally unhealthy, such as sugars and confectionary, sweetened beverages, cakes, and pastry, tend to be under-reported more often [39]. Foods eaten as snacks in-between meals are often significantly under-reported, even when main meals reports are accurate [40]. Due to these reasons, an increased awareness of the negative health effects arising from frequent consumption of sugary foods may have decreased the actual amount of free sugars consumed, but may have also prompted people to feel guiltier and thus less willing to report consumption. However, during the last 50 years, decreasing or at least stable dietary sugars consumption trends have been observed worldwide [36,41], not only in Slovenia. 

Across all age groups, women had a higher percentage of calories derived from sugars and free sugars compared to men, which is consistent with the majority of findings from the other European countries [35,36,42]. However, sex differences observed in the Slovenian population are more pronounced across all age groups. Among other sociodemographic parameters, level of education was negatively correlated with free sugars intake, which is in line with observations in France and The Netherlands [43]. Conversely, family net income was positively correlated with free sugars intake, which is the opposite of the trend reported in a Dutch population [35]. BMI and physical activity levels showed no direct correlations with the percentage of TEI consumed in the form of free sugars, which is in line with studies showing that energy intake but not total or added sugars intakes is a primary predictor of BMI [44].

The results of this study showed that fruit juices (12.6–19.7%); soft drinks (3.6–14%); cakes, muffins, and pastry (11–13.4%); and biscuits (6.3–8.5%) were the top dietary sources that contributed the most to free sugars intake. Adolescents and adults tended to consume more soft drinks, whereas elderly consumed more fruit juices, but also more jams and marmalade (8.3%) and honey (12.8%). Dairy products (10.4%) and chocolate and sweets (8.4%), but not jellies, were also notable sources of free sugars in adolescent population. The intake of total (intrinsic) sugars consumed with fresh fruits was twice as high in the elderly compared to adolescents, pointing to a generally low consumption of fresh fruit among younger participants. 

A recent review of dietary sources of added sugars across 10 European countries reported that the main sources of added sugars were sweet products (including cakes, biscuits, and jam), beverages, and dairy products [43], which is in line with our observation. However, the main sources of free sugars across different countries showed a certain degree of regional variability. In Slovenia, non-alcoholic beverages are by far the most important source of free sugars; the main sources in other European countries range from sweets and candy in The Netherlands [35], to cereals and cereal products in the U.K. [34]. However, direct comparisons of free sugars sources between different studies are difficult due to the different categorizations of food items. A study on a Swiss population is one of the few that used a very detailed categorization and is therefore more suitable for a direct comparison. Their results showed that sweet products and sweet spreads, including honey and jam (15%) in particular, are some of the major sources of free sugars in the Swiss population [42]. In contrast, Slovenian adolescents consumed only 9.9% of free sugars in the form of sweet spreads and jams combined, whereas in adults and the elderly, the proportion of free sugars from jams and honey combined was higher than in Switzerland (up to 21.1.%). Whereas in the Slovenian population, more free sugars come from fruit juices, people in Switzerland generally report higher intakes of soft drinks. Due to a well-preserved tradition of baking, cakes and biscuits combined represent one of the most important sources of free sugars in Slovenia regardless of age (20–21.3% compared to 8–12.4% in Switzerland) [42]. 

Adherence of the Slovenian population to the WHO guidelines to consume less than 10% TEI in free sugars was higher compared to that in other countries; more than half of the Slovenian adolescents and as many as 84% of adults and 81% of the elderly met the recommendation. In a similar study conducted in Switzerland, 36% of adolescents, 45% of adults, and 53% of the elderly reported free sugars consumption below 10% TEI [42], whereas in the U.K., the same criteria were met by 27% of adolescents and 54% of the adult participants [34]. Only Portugal has reported comparably high or even higher percentages of adherence to the guidelines than reported in the present study (51.3% among adolescents, 68.9–85.1% among adults, and 92.1% among the elderly) [45].

As in all similar studies, the major limitation of our study was use of two 24-h recalls as our approach, which is prone to shortfalls such as under- and mis-reporting. The success of this approach depends on several factors that cannot be sufficiently corrected, such as subject’s memory, cooperation, and communication skills. As the accuracy of the recall data can be substantially improved with well-trained interviewers [46], all our interviewers underwent a course in nutrition and nutritional surveillance in practice. Aside from the initial training, they were encouraged to contact one of the project leaders should a question or problem occur during the interview. Another limitation was the exclusion of children up to the age of 10, which prevents generalizing the findings for the entire Slovenian population. Lastly, it should be noted that due to occasional missing values for dietary fiber of certain foods in the OPEN food composition database, the energy content in these foods might be overestimated, and therefore the %TEI of (free) sugars intake is likely to be underestimated to some extent.

The main strengths of the present study include the robust pan-European methodological approach, a representative sample of Slovenian population (aged 10–74 years), and a complete and detailed nutritional composition database of pre-packaged foods on the Slovenian market, developed and updated specifically for this purpose [16]. Another strength of this study was that we looked not only into sugars consumption, dietary sources of sugars, and adherence to the guidelines, but also checked for socio-demographic and lifestyle parameters that could be correlated with free sugars consumption.

## 5. Conclusions

The present study revealed that with more than 80% participants habitually consuming less than 10% TEI in the form of free sugars, the majority of the Slovenian adult and elderly population adheres to the WHO guidelines on free sugars consumption. Although only about half of the adolescents meet the recommendation, the general trends in recent years appears to be promising. Additional efforts are needed to further decrease free sugars consumption among the teenage population, in which dietary patterns are still of the most concern. Future research should also address the youngest but the most vulnerable population of children under the age of 10 years.

## Figures and Tables

**Table 1:** Demographic characteristics of the study sample.

		Age Cohorts		
		Adolescents (10–17 Years)	Adults (18–64 Years)	Elderly (65–74 Years)
		*n* = 468 (%)	*n* = 364 (%)	*n* = 416 (%)
Age (mean (SD)) (years)		13.4 (2.37)	43.6 (13.81)	68.7 (2.7)
Place of living				
	Village	270 (57.7)	202 (55.5)	229 (55.1)
	Town	76 (16.2)	56 (15.4)	71 (17.1)
	City	122 (26.1)	106 (29.1)	116 (27.9)
Sex				
	Male	238 (50.9)	173 (47.5)	213 (51.2)
	Female	230 (49.1)	191 (52.5)	203 (48.8)
Education				
	No university degree	n.a.	249 (68.4)	342 (82.2)
	University degree	n.a.	115 (31.6)	74 (17.8)
Family monthly net income				
	Below average (≤1300 euros)	n.a.	118 (38.4)	269 (71.5)
	Above average (>1300 euros)	n.a.	189 (61.6)	107 (28.5)
BMI (mean ± SD)		21.0 (4.2)	26.7 (5.2)	28.4 (5.0)
	Normal	301 (64.6)	148 (40.7)	108 (26.0)
	Overweight and obese	167 (35.7)	216 (59.3)	308 (74.0)
IPAQ score				
	Low level	108 (23.3)	127 (35.3)	137 (33.4)
	Moderate level	141 (30.5)	108 (30.0)	133 (32.4)
	High level	214 (46.2)	125 (34.7)	140 (34.2)
Employment status				
	Employed	n.a.	226 (62.1)	n.a.
	Unemployed	n.a.	42 (11.5)	n.a.
	Student/pupil	n.a.	32 (8.8)	n.a.
	Retired	n.a.	64 (17.6)	n.a.

Note: Body mass index (BMI) was considered normal bellow 25 kg/m^2^, except for adolescents, where sex/age adjusted cut-off points [22,23] were used; SD, standard deviation; IPAQ, International Physical Activity Questionnaire; n.a., not applicable.

**Table 2:** Weighted habitual intakes of total sugars, free sugars, and carbohydrates (CHO).

		Total Sugars							Free Sugars								CHO				
	*n* (%)	Mean (g)	SE	CI Low	CI High	Median (g)	% TEI	% Total CHO	Mean (g)	SE	CI Low	CI High	Median (g)	% TEI	<10% TEI (%)	% Total CHO	Mean (g)	SE	CI Low	CI High	Median (g)
Adolescents	468	74.7	1.40	71.9	77.5	74.2	18.14	34.9	41.9	1.75	38.5	45.4	39.3	10.1	56.6	19.5	212.8	1.72	209.4	216.2	212.7
Male	238 (50.9)	73.5	2.40	68.8	78.3	74.2	15.9	34.2	41.6	3.18	35.4	47.9	39.3	8.9	65.4	19.2	213.4	3.06	207.4	219.4	213.4
Female	230 (49.1)	76.0	1.60	72.9	79.1	73.6	20.6	35.7	42.3	1.29	39.7	44.8	39.6	11.4	46.9	19.8	212.2	1.24	209.8	214.6	212.4
Adults	364	60.0	1.17	57.6	62.2	59.3	14.56	31.1	26.8	1.02	24.8	28.8	22.6	6.4	84.0	13.8	191.2	1.63	188.1	194.5	191.3
Male	173 (47.5)	56.7	1.91	52.9	60.4	56.9	12.1	29.6	27.6	1.66	24.3	30.9	20.7	5.8	88.8	14.3	189.3	1.62	184.2	194.4	185.9
Female	191 (52.5)	63.1	1.32	60.5	65.7	62.4	17.1	32.7	25.9	1.17	23.6	28.2	23.9	7.0	79.2	13.3	193.3	1.96	189.4	197.1	195.2
Elderly	416	64.4	1.72	61.0	67.8	65.0	15.87	33.0	27.0	2.31	22.5	31.6	21.8	6.5	81.0	13.8	191.9	3.56	184.9	198.9	190.1
Male	213 (51.2)	57.0	2.42	52.2	61.8	58.1	13.2	31.1	26.8	4.58	17.8	35.8	19.3	6.1	75.4	14.5	179.7	2.35	175.1	184.3	178.2
Female	203 (48.8)	71.2	2.20	66.9	75.5	70.4	18.3	34.7	27.3	1.48	24.4	30.2	25.1	6.9	85.8	13.2	203.1	5.16	193.0	213.2	202.8

SE, standard error; CI low, confidence interval-low; CI high, confidence interval-high; % TEI, percentage of total energy intake; % Total CHO, percentage of total carbohydrates; <10% TEI (%), percentage of participants adhering to WHO <10% recommendation for free sugars intake.

**Table 3:** Adjusted mean (95% CI) levels of free sugars (% TEI) intake by sex, place of living, BMI, IPAQ score, education, income, and employment for different age groups.

Variable		Adolescents (10–17 Years Old)		Adults (18–64 Years Old)		Elderly (65–74 Years Old)	
		*n* (%)	Adjusted	*n*	Adjusted	*n*	Adjusted
Overall		468 (37.5)		364 (29.2)		416 (33.3)	
Sex	Male	238 (50.9)	8.9 (8.2–9.5)	173 (47.5)	5.7 (4.9–6.4)	213 (51.2)	5.2 (4.6–5.9)
	Female	230 (49.1)	11.8 (11.1–12.4)	191 (52.5)	7.3 (6.6–8.0)	203 (48.8)	7.0 (6.4–7.7)
Place of living	Village	270 (57.7)	10.5 (9.9–11.1)	202 (55.5)	6.4 (5.7–7.1)	229 (55.1)	6.0 (5.3–6.6)
	Town	76 (16.2)	10.1 (9.0–11.2)	56 (15.4)	5.9 (4.7–7.3)	71 (17.1)	6.4 (5.3–7.5)
	City	122 (26.1)	10.1 (9.2–11.0)	106 (29.1)	6.9 (6.0–7.8)	116 (27.9)	6.2 (5.3–7.1)
Education	No university degree	n.a.	n.a.	249 (68.4)	7.0 (6.3–7.6)	342 (82.2)	6.2 (5.7–6.8)
	University degree			115 (31.6)	5.6 (4.6–6.5)	74 (17.8)	5.6 (4.4–6.7)
Family net income	Below average (≤1300 €)	n.a.	n.a.	118 (38.4)	5.8 (4.9–6.6)	269 (71.5)	6.2 (5.7–6.8)
	Above average (>1300 €)			189 (61.6)	7.0 (6.3–7.7)	107 (28.5)	5.8 (4.8–6.7)
BMI	Normal	301 (64.3)	10.4 (9.8–11.0)	148 (40.7)	6.8 (6.0–7.7)	108 (26.0)	5.7 (4.8–6.6)
	Overweight and obese	167 (35.7)	10.1 (9.0–11.3)	216 (59.3)	6.3 (5.8–6.9)	308 (74.0)	6.3 (5.7–6.8)
IPAQ	Low intensity	108 (23.3)	10.1 (9.2–11.1)	127 (35.3)	6.4 (5.5–7.3)	137 (33.4)	6.2 (5.4–7.0)
	Moderate	141 (30.5)	10.6 (9.8–11.5)	108 (30.0)	6.4 (5.5–7.3)	133 (32.4)	5.9 (5.1–6.7)
	High intensity	214 (46.2)	10.2 (9.5–10.9)	125 (34.7)	6.7 (5.9–7.6)	140 (34.2)	6.3 (5.5–7.1)
Employment	Employed	n.a.	n.a.	226 (62.1)	6.5 (5.9–7.2)	n.a.	n.a.
	Unemployed			42 (11.5)	7.6 (6.0–9.1)		
	Student			32 (8.8)	6.6 (4.6–8.6)		
	Retired			64 (17.6)	5.7 (4.4–7.0)		

Note: Body mass index (BMI) was considered as normal bellow 25 kg/m^2^, except for adolescents, where sex/age adjusted cut-off points [22,23] were used. Linear regression analysis conducted on samples with excluded missing values (Family net income: *n* = 57 (adults) and 40 (elderly); IPAQ: *n* = 5 (adolescents), 4 (adults), 6 (elderly)); Identified predictors accounting for variability in free sugars intake (%TEI): *p* < 0.001 sex (adolescents), *p* = 0.003 sex (adults), *p* = 0.002 sex (elderly), *p* = 0.024 education (adults), *p* = 0.041 family net income (adults).

**Table 4:** Mean contribution (%) to the total and free sugars intake by selected food groups.

Food Category	Food Sub-Category	Adolescents						Adults						Elderly					
		All		Boys		Girls		All		Men		Women		All		Men		Women	
		TS (%)	FS (%)	TS (%)	FS (%)	TS (%)	FS (%)	TS (%)	FS (%)	TS (%)	FS (%)	TS (%)	FS (%)	TS (%)	FS (%)	TS (%)	FS (%)	TS (%)	FS (%)
**Beverages**		20.7	32.7	22.0	34.1	18.8	30.7	18.8	34.1	23.2	38.4	14.0	27.7	16.6	31.4	23.2	41.1	11.9	23.2
	Alcoholic	0.2	0.2	0.2	0.3	0.1	0.1	0.9	1.4	1.1	1.8	0.6	0.9	0.8	1.4	1.6	2.5	0.3	0.5
	Coffee	0.2	0.1	0.1	0.1	0.3	0.2	2.0	1.5	1.9	1.4	2.2	1.7	2.8	2.8	3.0	2.9	2.7	2.7
	Cordials	0.6	1.0	0.7	1.1	0.4	0.7	0.4	0.8	0.6	1.1	0.2	0.4	0.2	0.4	0.2	0.5	0.1	0.3
	Energy drinks	0.4	0.7	0.7	1.0	0.1	0.2	0.4	0.7	0.6	1.1	0.1	0.2	0.0	0.0	0.0	0.0	0.0	0.0
	Instant coffee	0.1	0.2	0.0	0.1	0.3	0.4	0.6	1.2	0.5	0.8	0.7	1.7	0.5	1.1	0.4	0.8	0.6	1.4
	Soft drinks	7.4	11.7	8.0	12.2	6.6	10.9	7.2	14.0	9.2	15.8	5.1	11.4	1.7	3.6	2.0	4.0	1.4	3.2
	Tea	1.0	1.5	0.8	1.1	1.3	2.1	0.9	1.7	0.7	1.2	1.1	2.4	1.2	2.5	1.8	3.4	0.8	1.7
	Fruit and vegetable juices	10.6	17.1	11.3	17.9	9.6	16.0	6.3	12.6	8.4	15.0	3.9	9.0	9.1	19.7	13.7	27.0	5.8	13.5
**Bread and bakery products**		17.9	21.2	18.6	21.0	16.9	21.6	14.7	20.0	15.2	17.7	14.1	23.4	14.2	20.2	13.1	15.8	14.9	23.9
	Biscuits	5.4	8.5	6.2	9.4	4.3	7.2	4.3	8.3	4.9	8.5	3.6	8.1	3.0	6.3	3.5	6.8	2.6	5.9
	Bread	2.3	0.4	2.5	0.4	2.1	0.5	2.9	0.7	3.3	0.4	2.5	1.0	2.7	0.5	3.3	0.7	2.3	0.4
	Cakes, muffins, and pastry	10.2	12.3	9.9	11.2	10.5	14.0	7.4	11.0	6.9	8.8	8.0	14.4	8.4	13.4	6.2	8.3	10.0	17.6
	Dough	0.0	0.0	0.0	0.0	0.0	0.0	0.0	0.0	0.0	0.0	0.0	0.0	0.0	0.0	0.0	0.0	0.0	0.0
**Cereals and cereal products**		6.6	6.2	6.5	6.0	6.8	6.5	3.1	2.8	2.8	2.4	3.4	3.4	1.9	0.3	0.8	0.2	2.7	0.3
	Breakfast cereals	2.8	3.0	2.9	3.3	2.6	2.6	1.8	1.8	1.8	1.8	1.7	1.8	0.9	0.1	0.1	0.1	1.5	0.2
	Cereal bars	0.6	0.8	0.5	0.6	0.7	1.1	0.3	0.4	0.1	0.2	0.4	0.7	0.0	0.0	0.1	0.1	0.0	0.0
	Porridge	3.0	2.4	2.8	2.2	3.3	2.9	0.8	0.6	0.6	0.4	1.1	0.9	0.8	0.1	0.4	0.1	1.1	0.1
**Confectionery**		6.2	9.2	6.1	8.9	6.4	9.8	3.3	6.2	4.0	6.7	2.6	5.4	1.7	3.4	2.1	3.8	1.4	3.0
	Chocolate and sweets	5.7	8.4	5.7	8.4	5.6	8.5	3.3	6.1	4.0	6.6	2.5	5.3	1.6	3.2	1.9	3.5	1.4	3.0
	Jelly	0.5	0.8	0.3	0.5	0.7	1.2	0.0	0.0	0.0	0.0	0.0	0.1	0.1	0.1	0.2	0.3	0.0	0.0
**Dairy**		16.3	10.7	15.7	9.2	17.2	12.8	14.3	10.3	12.7	8.5	16.2	13.0	10.3	5.1	8.8	4.2	11.4	5.9
	Desserts	0.9	1.0	0.9	1.0	1.0	1.0	1.4	2.2	1.5	2.3	1.2	2.2	0.5	0.7	0.5	0.7	0.6	0.7
	Flavored yoghurt	2.8	3.1	2.7	2.8	3.0	3.4	2.4	3.1	2.4	2.8	2.3	3.6	1.0	1.4	0.7	0.8	1.3	2.0
	Ice cream and edible ices	2.9	4.0	2.4	3.1	3.6	5.2	2.0	3.3	1.1	1.7	3.0	5.7	1.1	1.9	1.0	1.6	1.2	2.2
	Milk	5.5	0.0	5.7	0.0	5.2	0.0	4.1	0.0	3.7	0.0	4.7	0.0	3.1	0.0	2.9	0.0	3.3	0.0
	Milk drinks	1.5	2.0	1.4	1.7	1.8	2.4	0.5	0.8	0.8	1.1	0.3	0.5	0.2	0.3	0.1	0.2	0.2	0.4
	Plain yoghurt	1.8	0.3	1.6	0.2	2.0	0.3	3.3	0.6	2.7	0.4	3.9	0.9	3.9	0.7	3.2	1.0	4.4	0.5
**Fruit and vegetable products**		19.3	7.0	17.7	7.6	21.5	6.0	29.4	7.8	24.5	7.9	34.8	7.6	38.7	16.1	31.3	8.0	43.9	22.9
	Canned fruit	1.0	1.1	1.2	1.3	0.7	0.7	1.4	2.2	2.0	2.8	0.7	1.5	5.6	6.0	1.3	1.8	8.7	9.5
	Dried fruit	0.7	0.0	0.4	0.0	1.0	0.1	1.0	0.0	0.6	0.0	1.5	0.0	1.8	0.2	1.2	0.0	2.2	0.4
	Fresh fruit	11.9	0.1	10.2	0.1	14.3	0.1	20.0	0.2	15.3	0.3	25.2	0.2	23.6	1.6	22.3	0.9	24.5	2.1
	Jam and marmalade	4.0	5.7	4.4	6.2	3.4	5.0	3.1	5.2	3.3	4.9	3.0	5.7	4.6	8.3	3.5	5.2	5.4	10.8
	Nuts and fruit mixes	0.4	0.0	0.2	0.0	0.7	0.0	0.9	0.0	0.7	0.0	1.1	0.0	0.3	0.0	0.4	0.0	0.2	0.0
	Raw vegetables	0.7	0.0	0.8	0.0	0.6	0.0	1.5	0.0	1.2	0.0	1.8	0.0	1.4	0.0	1.4	0.0	1.5	0.0
**Sauces and spreads**		3.7	5.0	3.5	4.5	3.9	5.8	1.7	2.5	2.4	3.2	0.9	1.4	0.5	0.4	0.7	0.5	0.4	0.4
	Sweet spreads	3.0	4.4	2.7	3.7	3.5	5.5	0.9	1.7	1.3	2.1	0.5	1.1	0.1	0.1	0.2	0.3	0.0	0.0
**Sugars, honey, and related products**		3.7	6.1	4.1	6.4	3.3	5.6	7.0	14.0	7.1	12.6	6.8	15.9	10.0	21.8	12.6	24.9	8.1	19.1
	Honey	1.1	1.8	1.2	1.9	1.0	1.7	3.0	5.9	1.9	3.4	4.2	9.7	5.9	12.8	6.6	13.0	5.3	12.5
	Sugar	1.1	1.8	0.9	1.4	1.4	2.4	3.0	6.1	3.5	6.3	2.5	5.7	2.9	6.2	3.7	7.3	2.3	5.3
	Syrup	1.5	2.5	2.0	3.1	0.9	1.5	1.0	2.0	1.7	3.0	0.2	0.5	1.3	2.8	2.3	4.6	0.5	1.3

TS, total sugars; FS, free sugars.

**Table 5:** Percentage of the population meeting recommendations for free sugars intake (<10% total energy intake) by sex, place of living, education, family net income, BMI, IPAQ, and employment.

Variable		Adolescents (10–17 Years Old)			Adults (18–64 Years Old)			Elderly (65–74 Years Old)		
		*n*	<10% TEI *n* (%)	Odds Ratio *	*n*	<10% TEI *n* (%)	Odds Ratio *	*n*	<10% TEI *n* (%)	Odds Ratio *
Overall		468	262 (56.0)		364	299 (82.1)		416	356 (85.6)	
Sex	Male	238	164 (68.9)	1	173	153 (88.4)	1	213	189 (88.7)	1
	Female	230	98 (42.6)	0.32 (0.21–0.48)	191	146 (76.4)	0.40 (0.21–0.79)	203	167 (82.3)	0.61 (0.33–1.14)
Place of living	Village	270	144 (53.3)	1	202	166 (82.2)	1	229	196 (85.6)	1
	Town	76	42 (55.3)	1.15 (0.68–1.96)	56	46 (82.1)	1.59 (0.60–4.17)	71	60 (84.5)	0.93 (0.43–2.00)
	City	122	76 (62.3)	1.41 (0.88–2.22)	106	87 (82.1)	0.91 (0.46–1.82)	116	100 (86.2)	1.30 (0.62–2.70)
Education	No university degree		n.a.	n.a.	249	202 (81.1)	1	342	292 (85.4)	1
	University degree				115	97 (87.4)	1.75 (0.84–3.70)	74	64 (86.5)	1.05 (0.44–2.5)
Family net income	Below average (≤1300 €)		n.a.	n.a.	118	103 (87.3)	1	269	230 (85.5)	1
	Above average (>1300 €)				189	150 (79.4)	0.45 (0.21–0,96)	107	94 (87.9)	1.20 (0.59–2.44)
BMI	Normal	301	165 (54.8)	1	148	120 (81.1)	1	108	92 (85.2)	1
	Overweight and obese	167	97 (58.1)	1.05 (0.71–1.59)	216	179 (82.3)	1.10 (0.56–2.17)	308	264 (85.7)	0.92 (0.45–1.89)
IPAQ	Low intensity	108	59 (54.63)	1	127	103 (81.1)	1	137	117 (85.4)	1
	Moderate	141	78 (55.3)	1.16 (0.68–2.00)	108	94 (87.0)	1.59 (0.70–3.57)	133	112 (84.2)	1.04 (0.51–2.13)
	High intensity	214	124 (57.9)	1.16 (0.71–1.89)	125	99 (79.2)	0.90 (0.43–1.85)	140	121 (86.4)	1.10 (0.53–2.27)
Employment	Employed		n.a.	n.a.	226	184 (81.4)	1		n.a.	n.a.
	Unemployed				42	33 (78.6)	0.48 (0.18–1.28)			
	Student				32	26 (81.3)	0.92 (0.28–2.94)			
	Retired				64	56 (87.5)	1.56 (0.56–4.35)			

Note: Body mass index (BMI) was considered as normal bellow 25 kg/m^2^, except for adolescents, where sex/age adjusted cut-off points [22,23] were used. Logistic regression analysis conducted on samples with excluded missing values (Family net income: *n* = 57 (adults) and 40 (elderly); IPAQ: *n* = 5 (adolescents), 4 (adults), 6 (elderly); * Odds ratio formeeting free sugars intake recommendations; Variables, significantly correlated with the adherence to WHO recommendation: *p* = 0.001 sex (adolescents), *p* = 0.008 sex (adults), *p* = 0.038 family net income (adults).

## Supplementary Materials

The following are available online at https://www.mdpi.com/2072-6643/12/6/1729/s1. Table S1: Unweighted adjusted and unadjusted mean (95% CI) levels of free sugars (% TEI) intake by sex, place of living, BMI, IPAQ score, education, income, and employment for different age groups, Table S2: Mean contribution (%) to the total and free sugars intake per food groups (all), Table S3: Habitual intakes of total, free, and CHO TEI: total energy intake and percent of total CHO per age categories and different individual and sociodemographic groups.
